# *Roseburia hominis* Increases Intestinal Melatonin Level by Activating p-CREB-AANAT Pathway

**DOI:** 10.3390/nu14010117

**Published:** 2021-12-28

**Authors:** Lijin Song, Meibo He, Qinghua Sun, Yujing Wang, Jindong Zhang, Yuan Fang, Shuangjiang Liu, Liping Duan

**Affiliations:** 1 Department of Gastroenterology, Peking University Third Hospital, Beijing 100191, China; songlj851@163.com (L.S.); sunqinghua@bjmu.edu.cn (Q.S.); zhangjd@bjmu.edu.cn (J.Z.); fsz252@163.com (Y.F.); 2 Institute of Systems Biomedicine, School of Basic Medical Sciences, Peking University Health Science Center, Beijing 100191, China; iris0402@foxmail.com; 3 State Key Laboratory of Microbial Resources, Institute of Microbiology, Chinese Academy of Sciences, Beijing 100101, China; wangyujinng15@mails.ucas.edu.cn (Y.W.); liusj@im.ac.cn (S.L.)

**Keywords:** *Roseburia hominis*, melatonin, butyrate, propionate, 5-HT, AANAT, CREB

## Abstract

Intestinal melatonin exerts diverse biological effects on the body. Our previous research showed that the abundance of the butyrate-producing bacteria, *Roseburia*, is positively related to the expression of colonic mucosal melatonin. However, the detailed relationship is unclear. Therefore, we aimed to explore whether *Roseburia* regulates intestinal melatonin and its underlying mechanisms. Male Sprague–Dawley germfree rats were orally administered with or without *Roseburia hominis*. *R. hominis* treatment significantly increased the intestinal melatonin level. The concentrations of propionate and butyrate in the intestinal contents were significantly elevated after gavage of *R. hominis*. Propionate or butyrate treatment increased melatonin, 5-hydroxytryptamine (5-HT), arylalkylamine N-acetyltransferase (AANAT), and phosphorylated cAMP-response element-binding protein (p-CREB) levels. When pretreated with telotristat ethyl, the inhibitor of tryptophan hydroxylase (TPH), or siRNA of *Aanat*, or 666-15, i.e., an inhibitor of CREB, propionate, or butyrate, could not promote melatonin production in the pheochromocytoma cell line BON-1. Metabolomics analysis showed that propionate and butyrate stimulation regulated levels of some metabolites and some metabolic pathways in BON-1 cell supernatants. In conclusion, propionate and butyrate, i.e., metabolites of *R. hominis*, can promote intestinal melatonin synthesis by increasing 5-HT levels and promoting p-CREB-mediated *Aanat* transcription, thereby offering a potential target for ameliorating intestinal diseases.

## 1. Introduction

Melatonin is generally considered a pineal hormone that can maintain circadian rhythms and regulate immune function. Melatonin is also distributed in the gastrointestinal (GI) tract [1], fulfilling vital antioxidant and anti-inflammatory functions locally and regulating gut motility. Melatonin supplementation alleviates the symptoms of digestive disorders, such as irritable bowel syndrome (IBS) and ulcerative colitis [2]. However, augmentation of the melatonin concentration in blood by oral administration may cause some adverse effects, including sleepiness, nausea, dizziness, and headaches [3]. As melatonin is a systemic hormone, it is desirable to find a way to locally induce melatonin synthesis to avoid adverse effects or the risk of hormone disturbance, which occurs when it is used for extended periods for the treatment of digestive disorders.

The intestinal microbiota contributes to colonic melatonin expression, as revealed in our previous study [4]. Furthermore, the abundance of *Roseburia*, a butyrate-producing genus, is positively related to colonic mucosal melatonin level [4]. *Roseburia* is Gram-positive anaerobic bacteria, including five species: *Roseburia intestinalis*, *R. hominis*, *R. inulinivorans*, *R. faecis*, and *R. cecicola*. Short-chain fatty acids (SCFAs) are important metabolites of *Roseburia*, derived from the bacterial fermentation of dietary fibers [5]. However, whether *Roseburia* modulates intestinal melatonin expression and the underlying mechanisms remains unclear. Gut melatonin is synthesized mainly by enterochromaffin cells (ECs) in the GI tract, and its synthesis is influenced by its upstream product, 5-hydroxytryptamine (5-HT). A recent study showed that indigenous spore-forming bacteria promote 5-HT biosynthesis from colonic ECs. Furthermore, certain microbiota metabolites, such as propionate and butyrate, could elevate 5-HT levels in chromaffin cell cultures [6]. These findings imply that 5-HT plays an essential role in the effect of gut microbiota on melatonin. In addition, arylalkylamine N-acetyltransferase (AANAT) is a rate-limiting enzyme in melatonin synthesis that notably regulates melatonin levels [7]. However, whether *Roseburia* could regulate intestinal melatonin synthesis by elevating 5-HT production and/or promoting *Aanat* transcription is ambiguous.

In this study, germfree rats and the EC model BON-1 were utilized to determine the relationship between *Roseburia* and melatonin, as well as the specific mechanism involving propionate, butyrate, 5-HT, and the phosphorylated cAMP-response element-binding protein (p-CREB)-AANAT pathway. Investigating these aspects may provide a new therapeutic strategy for IBS or inflammatory bowel syndrome.

## 2. Materials and Methods

### 2.1. Animals and Experimental Design

Male germfree Sprague–Dawley rats (7 weeks old) were obtained from the Department of Laboratory Animal Science at Peking University Health Science Center, Beijing, China. Germfree rats were housed in sterilized isolators and maintained under a 12-h light/dark cycle (6 A.M. to 6 P.M.) at a constant temperature (23 ± 2 °C) and humidity (63 ± 2%). Food and water were provided ad libitum. All animals were acclimatized to the facility for 7 days before the experiment began. In the first experiment, germfree rats were orally administered with *Roseburia hominis* (2 × 10^9^ CFU/day) or phosphate-buffered saline (PBS) for 5 days (*n* = 6/group). *R. hominis* was prepared as previously described [8]. After colonization for 14 days, the visceral sensitivity was assessed using abdominal withdrawal reflex (AWR) score to colorectal distension as previously described [8]: 0, no behavioral response; 1, brief head movement followed by immobility; 2, contraction of abdominal muscles; 3, lifting of abdomen; and 4, body arching and lifting of pelvic structures. Then, the rats were anesthetized and sacrificed by intraperitoneal injection of 1% pentobarbital sodium. The intestinal contents were collected for short-chain fatty acids (SCFAs) analysis. The intestinal tissue was fixed in 10% buffered formalin for immunohistochemistry (IHC) staining. Serum was collected for melatonin measurements. In the second experiment, germfree rats were orally administered sodium propionate (#P1880, Sigma-Aldrich, St. Louis, MO, USA, 300 mg/kg/day, Group GP), sodium butyrate (#303410, Sigma-Aldrich, 300 mg/kg/day, Group GB), or PBS (Group GF) of the same volume for 7 days (*n* = 6/group). Rats were anesthetized and sacrificed by intraperitoneal injection of 1% pentobarbital sodium after gavage. Intestinal tissue was collected for IHC, Western blotting, enzyme-linked immunosorbent assay (ELISA), and quantitative polymerase chain reaction (qPCR). Serum was collected for melatonin measurements. All the animals fasted overnight and were sacrificed from 9 A.M. to 12 P.M. All protocols were approved by the Laboratory Animal Welfare Ethics branch of the Biomedical Ethics Committee of Peking University (Approval No. LA2020509).

### 2.2. Targeted SCFAs Measurements

The SCFAs were extracted from the intestinal contents, according to the manufacturer’s instructions (Majorbio Bio-Pharm Technology Co., Ltd., Shanghai, China), and assayed using an 8890B-5977B gas chromatography-mass spectrometry detection system (Agilent Technologies, Santa Clara, CA, USA) as previously described [9].

### 2.3. Immunohistochemistry (IHC)

Distal ileum and colon tissues were embedded in paraffin and cut into 5-μm-thick sections. After deparaffinization and rehydration, endogenous peroxidase was removed with 3% hydrogen peroxide for 10 min. The sections were then incubated with primary melatonin (1:200, #abx100179, Abbexa, Cambridge, UK) or 5-HT (1:100, #PAA808Ge01, Cloud Clone, Wuhan, China) antibodies. The sections were washed with PBS and incubated with a horseradish peroxidase (HRP)-conjugated secondary antibody (#PV6001, ZSGB-BIO, Beijing, China). Images were captured using a NanoZoomer-SQ Digital Pathology scanner (Hamamatsu Photonics, Hamamatsu City, Japan) and exported using NDP.view software (version 2.6.8, Hamamatsu Photonics, Hamamatsu City, Japan). For the quantification of melatonin and 5-HT levels, five fields were randomly selected from each slide at a magnification of 400×. The number of immunoreactive cells was counted in every field, and the density of immunoreactive cells was expressed as the average number of cells per square millimeter of the mucosal epithelium.

### 2.4. Cell Culture

BON-1 cells were purchased from Shanghai Shun Ran Biotechnology Co., Ltd. (Shanghai, China), and cultured in Dulbecco’s Modified Eagle Medium/Nutrient Mixture F-12 (DMEM/F12) (HyClone, Logan, UT, USA) containing 10% fetal bovine serum (FBS, Gibco, Grand Island, NY, USA) and 1% penicillin/streptomycin (Gibco) at 37 °C in a humidified atmosphere of 5% CO_2_ [10]. BON-1 cells were seeded in six-well plates (1 × 10^6^ cells/well) and cultured without serum for 12 h before treatment. BON-1 cells were treated with bacterial medium, *R. hominis* supernatant, PBS, sodium propionate (#P1880, Sigma Aldrich), or sodium butyrate (#303410, Sigma Aldrich) for 24 h. The total protein and RNA and supernatant were extracted for further analysis.

BON-1 cells were pretreated with telotristat ethyl (inhibitor of tryptophan hydroxylase, #HY-13055A, MedChemExpress, Monmouth Junction, NJ, USA) and 666-15 (CREB inhibitor, #HY-101120, MedChemExpress, Monmouth Junction, NJ, USA), followed by treatment with sodium propionate and sodium butyrate [11]. BON-1 cells were transfected with *Aanat* small interfering RNA (siRNA, #sc-61928, Santa Cruz, CA, USA) using a transfection reagent (#301704, Qiagen, Hilden, Germany), according to the manufacturer’s instructions. The cells were then treated with sodium propionate or sodium butyrate for another 24 h. The total protein and supernatant were extracted for further detection.

### 2.5. ELISA

Intestinal tissue (100 mg) from rats was homogenized in ice-cold PBS (0.01 M, pH = 7.4). The supernatant was collected after centrifugation at 3000 rpm for 20 min at 4 °C, and the concentration of 5-HT was quantified using an ELISA kit (CEA808Ge, Cloud Clone, Wuhan, China), according to the manufacturer’s recommendations. The 5-HT level in BON-1 cell lysate was also detected using ELISA. The supernatant from BON-1 cells was collected, and the concentration of melatonin was measured using an ELISA kit (E-EL-H2016c, Elabscience, Wuhan, China). Absorbance was measured at 450 nm (Spark, Tecan, Switzerland). All values were in the linear range, and readings were normalized to the total protein content.

### 2.6. Serum Melatonin Measurements

Blood was withdrawn from the apex cordis of the rats after euthanasia. The supernatant was collected after centrifugation at 3000 rpm for 10 min at 4 °C and stored at −80 °C until analysis. High-performance liquid chromatography, coupled to tandem mass spectrometry (HPLC-MS/MS), was used to determine the melatonin concentrations according to the manufacturer’s instructions.

### 2.7. Western Blotting

Total protein was extracted from the intestinal tissue or BON-1 cells in radioimmunoprecipitation assay (RIPA) extraction buffer mixed with a protease inhibitor cocktail, and then centrifuged at 12,000 rpm at 4 °C for 10 min. Protein concentration was determined using a bicinchoninic acid assay. Protein samples (60 μg) were separated using 12% sodium dodecyl sulfate polyacrylamide gels and transferred onto nitrocellulose membranes. Membranes were blocked with 5% (*w*/*v*) dried skim milk at 22 °C for 1 h, and then incubated overnight at 4 °C with primary antibodies, including AANAT (1:1000, #ab3505, Abcam, Cambridge, UK), CREB (1:1000, #9197, Cell Signaling Technology, Danvers, MA, USA), phosphorylated CREB (p-CREB; Ser133, 1:1000, #9198, Cell Signaling Technology, Danvers, MA, USA), and glyceraldehyde-3phosphate dehydrogenase (GAPDH, 1:1000, #5174, Cell Signaling Technology, Danvers, MA, USA) antibodies. Subsequently, membranes were incubated with the corresponding secondary HRP-conjugated antibodies for 1 h at room temperature and then visualized using Immobilon Western Chemiluminescent HRP Substrate (Millipore Corporation, Burlington, MA, USA). Blotting images were obtained using a chemiluminescence detection system (Tanon, Shanghai, China) and quantified by calculating the gray value of each band using ImageJ (version 1.52) software. The levels were determined as the band intensity relative to that of GAPDH.

### 2.8. qPCR

Total RNA was extracted from the intestinal tissue or BON-1 cells using TRIzol reagent (Invitrogen, Carlsbad, CA, USA), according to the manufacturer’s instructions. Complementary DNA was synthesized using a Reverse Transcription Kit (Tiangen, Beijing, China). qPCR for *Aanat* and β-actin was performed using a Real-Time PCR Detection System (QuantStudio5, Thermo Fisher Scientific, Waltham, MA, USA) using SYBR Green. Primer sequences used for qPCR were as follows: forward *Aanat*, 5′-AAAGTACACTCAGGCACCAATGT-3′; reverse *Aanat*, 5′-GGGAACATAGCTGCTTTATTAGTGTCAG-3′; forward β-actin, 5′-GGGAAATCGTGCGTGACATT-3′; and reverse β-actin, 5′-GCGGCAGTGGCCATCTC-3′. PCR amplifications were performed in a total volume of 20 μL containing Talent qPCR PreMix (Tiangen, Beijing, China). The gene expression level was calculated using the 2^−ΔΔCt^ method, and the relative *Aanat* mRNA level was normalized to that of β-actin.

### 2.9. Non-Target Metabolomics

BON-1 cell supernatant was acquired using an ultra-high-performance liquid chromatography-mass spectrometry system following the manufacturer’s instructions. Non-target metabolomics of the supernatant was performed as previously described [12]. The raw data were converted to the mzXML format using ProteoWizard, which was developed using R and based on XCMS, for peak detection, extraction, alignment, and integration. The Human Metabolome Database (HMDB) was then used for metabolite annotation. The cutoff for annotation was set at 0.3.

### 2.10. Statistical Analysis

For parametric data, one-way analysis of variance (ANOVA) with Tukey’s post-hoc test for two sides was performed when more than two groups were evaluated. An unpaired Student’s *t*-test was used when there were two groups. For nonparametric data, a Kruskal–Wallis ANOVA combined with post-hoc Dunn’s multiple comparison test for two sides was performed when more than two groups were evaluated, and a Mann–Whitney test was performed when there were two groups. Results are expressed as the mean ± standard error of the mean (SEM). Differences between groups were considered significant at *p* < 0.05. Statistical analyses were performed using GraphPad Prism software (version 8.0; GraphPad Software Inc., San Diego, CA, USA).

## 3. Results

### 3.1. R. Hominis Increased Melatonin Level in the Rat Intestine and BON-1 Cells

**Figure 1:**
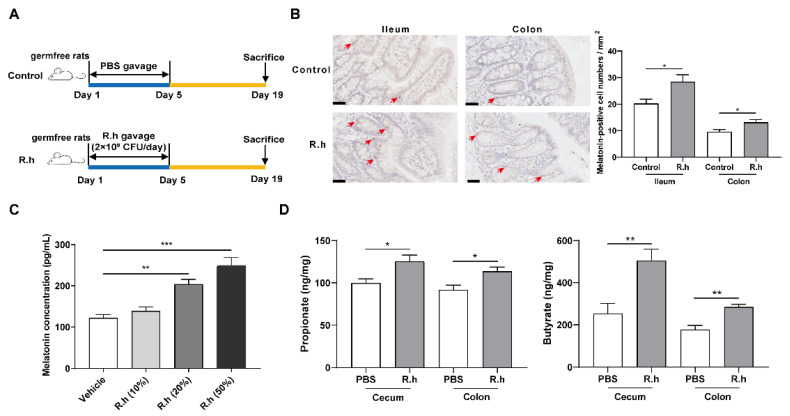
*Roseburia hominis* increased melatonin both in vivo and in vitro. (**A**) Schematic of the animal experiments. (**B**) Representative images (left, 400×) and quantification of immunohistochemistry (IHC) staining (right) for melatonin-positive cells in the ileal and colonic mucosa after *R. hominis* (R.h) was administered to germfree rats (*n* = 6; scale bars: 50 μm). Arrows indicate melatonin-positive cells according to the Mann–Whitney test. (**C**) Effects of different proportions (10%, 20%, and 50%) of R.h culture supernatant on the melatonin concentration in BON-1 cell supernatant detected using ELISA. Analysis was performed using one-way ANOVA with Tukey’s post-hoc test (*n* = 6). (**D**) Concentrations of propionate and butyrate in intestinal contents detected using gas chromatography–mass spectrometry detection system after gavage of R.h. Analysis was performed using unpaired Student’s *t*-test (*n* = 6). * *p* < 0.05, ** *p* < 0.01, *** *p* < 0.001.

To determine the effect of *R. hominis* on intestinal melatonin, germfree rats were treated with R. hominis or PBS. Oral administration of R. hominis at 2 × 10^9^ CFU/day for 5 days significantly increased the number of melatonin-positive cells in both the ileal and colonic mucosa compared to that in the control group (*p* < 0.05, Figure 1B). When treated with 20% and 50% proportions of *R. hominis* supernatant for 24 h, the concentration of melatonin in BON-1 cell supernatant was significantly elevated compared to that in the control group, as detected using ELISA (*p* < 0.05, Figure 1C). In addition, *R. hominis* administration increased the concentrations of propionate and butyrate both in the cecum and colon contents, as detected using targeted metabolomics (*p* < 0.05, Figure 1D). Meanwhile, other kinds of SCFAs, including acetate, isobutyrate, and isovalerate, did not show significant variation (Supplementary Figure S1).

### 3.2. Propionate and Butyrate Increased Melatonin Level in the Rat Intestine and BON-1 Cells

**Figure 2:**
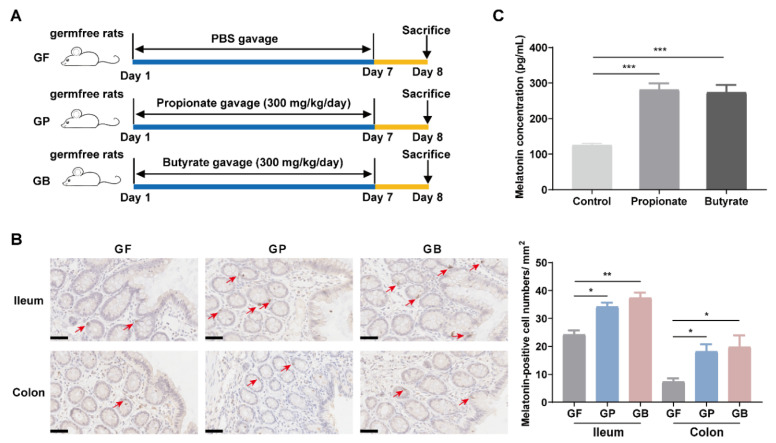
Propionate and butyrate increased melatonin levels both in vivo and in vitro. (**A**) Schematic of the animal experiments. (**B**) Representative images (left, 400×) and quantification of immunohistochemistry (IHC) staining (right) for melatonin-positive cells in the ileal and colonic mucosa after sodium propionate or sodium butyrate were administered to germfree rats (*n* = 6; scale bars: 50 μm). Arrows indicate melatonin-positive cells. Analysis was performed using Kruskal–Wallis test with Dunn’s multiple comparison post-hoc test. (**C**) Effects of sodium propionate (10 mM) or sodium butyrate (10 mM) on the concentration of melatonin in BON-1 cell supernatant detected using ELISA. Analysis was performed using one-way ANOVA with Tukey’s post-hoc test (*n* = 6). GF, germfree rats receiving PBS gavage; GP, germfree rats receiving propionate gavage; GB, germfree rats receiving butyrate gavage. * *p* < 0.05, ** *p* < 0.01, *** *p* < 0.001.

To clarify the effects of propionate and butyrate produced by *R. hominis* on melatonin levels, germfree rats and BON-1 cells were treated with propionate or butyrate. Oral administration of sodium propionate (300 mg/kg/day) or sodium butyrate (300 mg/kg/day) for 7 consecutive days significantly increased the density of melatonin-positive cells in the ileal and colonic mucosa compared to that in the control group (*p* < 0.05, Figure 2B). Propionate and butyrate treatment also improved the visceral hypersensitivity of germfree rats, and there is significant negative correlation between visceral sensitivity and intestinal melatonin level (Supplementary Figure S2). Treatment of BON-1 cells with sodium propionate (10 mM) or sodium butyrate (10 mM) increased the melatonin concentration in the supernatant (*p* < 0.05, Figure 2C).

### 3.3. Propionate and Butyrate Increased Melatonin Level by Promoting 5-HT Production

**Figure 3:**
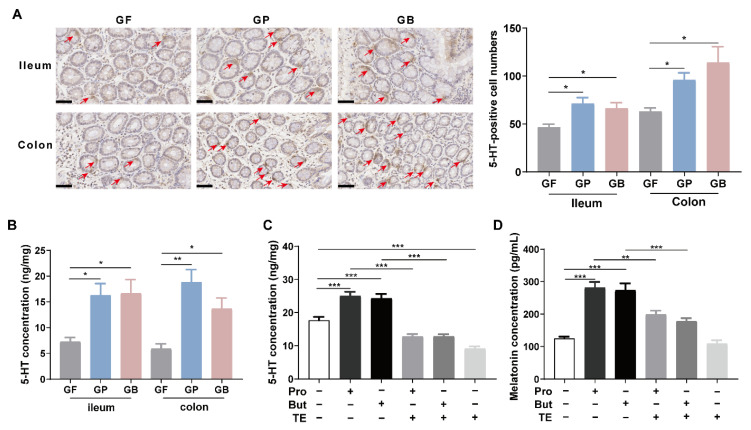
Propionate and butyrate increased melatonin level by promoting 5-hydroxytryptamine (5-HT) production. (**A**) Representative images (left, 400×) and quantification of immunohistochemistry (IHC) staining (right) for 5-HT-positive cells in the ileal and colonic mucosa after sodium propionate or sodium butyrate were administered to germfree rats (*n* = 6; scale bars: 50 μm). Arrows indicate 5-HT-positive cells. Analysis was performed using Kruskal–Wallis test with Dunn’s multiple comparison post-hoc test. (**B**) Intestinal 5-HT concentrations detected using ELISA. Analysis was performed using one-way ANOVA with Tukey’s post-hoc test (*n* = 6). (**C**) 5-HT concentration in BON-1 cell lysates detected using ELISA. Analysis was performed using one-way ANOVA with Tukey’s post-hoc test (*n* = 6). (**D**) Melatonin concentration in BON-1 cell supernatant after sodium propionate/sodium butyrate (10 mM) treatment for 24 h with or without telotristat ethyl (TE, 1 μM) pretreatment for 2 h, as detected using ELISA. Analysis was performed using one-way ANOVA with Tukey’s post-hoc test (*n* = 6). GF, germfree rats receiving PBS gavage; GP, germfree rats receiving propionate gavage; GB, germfree rats receiving butyrate gavage. * *p* < 0.05, ** *p* < 0.01, *** *p* < 0.001.

To determine the mechanism by which propionate and butyrate increased intestinal melatonin level, 5-HT levels were measured after treatment with propionate or butyrate. Oral administration of sodium propionate (300 mg/kg/day) or sodium butyrate (300 mg/kg/day) for 7 days increased the number of 5-HT-positive cells in the ileal and colonic mucosa, as detected using IHC staining (*p* < 0.05, Figure 3A). The same results were obtained using ELISA (Figure 3B). When treated with sodium propionate (10 mM) or sodium butyrate (10 mM) for 24 h, the 5-HT concentration in the BON-1 cell lysate was significantly elevated compared with that in the control group, as detected using ELISA (*p* < 0.05, Figure 3C). Telotristat ethyl is an inhibitor of tryptophan hydroxylase (TPH), which is the rate-limiting enzyme in 5-HT biosynthesis [13]. Pretreatment with telotristat ethyl (1 μM) for 2 h prevented the increase in 5-HT level caused by propionate or butyrate administration (Figure 3C). Telotristat ethyl treatment also inhibited the increase in melatonin levels (Figure 3D).

### 3.4. Propionate and Butyrate Increased Melatonin Level through the p-CREB-AANAT Pathway

**Figure 4:**
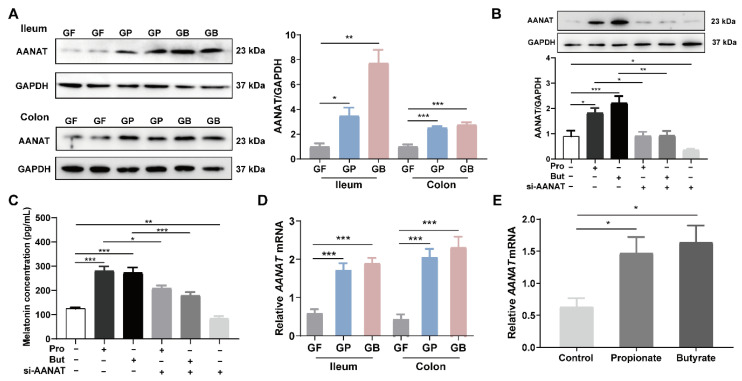
Propionate and butyrate increased intestinal AANAT level in rats. (**A**) Representative images (left) and quantification of intestinal AANAT level (right) detected using Western blotting after administration of sodium propionate or sodium butyrate to germfree rats. Analysis was performed using Brown–Forsythe and Welch ANOVA test with Tamhane’s T2 post-hoc test (*n* = 6). (**B**) Representative images (above) and quantification of AANAT level (below) in BON-1 cells after propionate/butyrate (10 mM) treatment for 24 h with or without *Aanat* siRNA treatment for 32 h. Results were obtained using Western blotting and analyzed using one-way ANOVA with Tukey’s post-hoc test. *n* = 6. (**C**) Melatonin concentration in BON-1 cell supernatant after propionate/butyrate (10 mM) treatment for 24 h with or without *Aanat* siRNA treatment for 32 h. Results were detected using ELISA and analyzed using one-way ANOVA with Tukey’s post-hoc test (*n* = 6). (**D**) Intestinal *Aanat* mRNA expression normalized to β-actin expression after administration of sodium propionate or sodium butyrate to germfree rats. Results were obtained using qPCR and analyzed using one-way ANOVA with Tukey’s post-hoc test (*n* = 6). (**E**) *Aanat* mRNA expression normalized to β-actin expression in BON-1 cells after treatment with propionate/butyrate (10 mM) for 24 h. Analysis was performed using one-way ANOVA with Tukey’s post-hoc test (*n* = 6). GF, germfree rats receiving PBS gavage; GP, germfree rats receiving propionate gavage; GB, germfree rats receiving butyrate gavage. * *p* < 0.05, ** *p* < 0.01, *** *p* < 0.001.

Oral administration of sodium propionate (300 mg/kg/day) or sodium butyrate (300 mg/kg/day) for 7 days significantly increased AANAT levels in rat intestinal tissue, as detected using Western blotting (*p* < 0.05, Figure 4A). Consistently, treatment of BON-1 cells with sodium propionate (10 mM) or sodium butyrate (10 mM) for 24 h significantly increased the AANAT level following Western blotting and ELISA (*p* < 0.05, Figure 4B,C). Besides, the promoting effect of propionate and butyrate on AANAT level is in a time-dependent manner (Supplementary Figure S3). Pretreatment with small interfering RNA (siRNA) of *Aanat* reduced AANAT and melatonin levels in BON-1 cells (*p* < 0.05, Figure 4B,C). Moreover, the *Aanat* mRNA level was notably elevated when treated with propionate and butyrate, both in vivo and in vitro (*p* < 0.05, Figure 4D,E). 

**Figure 5:**
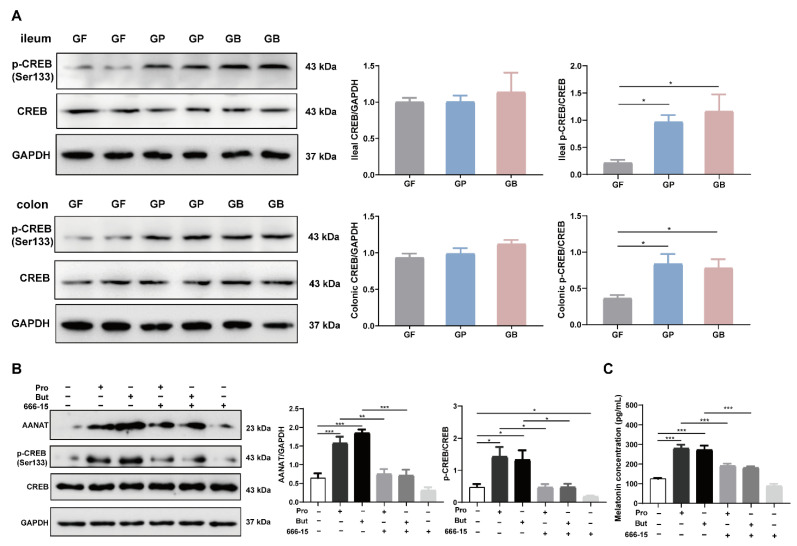
Propionate and butyrate increased AANAT and melatonin levels mediated by p-CREB. (**A**) Representative images (left) and quantification of intestinal p-CREB and CREB (right) levels detected using Western blotting after administration of sodium propionate or sodium butyrate to germfree rats (*n* = 6). (**B**) Representative images (left) and quantification of AANAT and p-CREB (right) levels in BON-1 cells detected using Western blotting after propionate/butyrate (10 mM) treatment for 24 h with or without 666-15 pretreatment (1 μM) for 2 h (*n* = 6). (**C**) Melatonin concentration in BON-1 cell supernatant detected using ELISA after propionate/butyrate (10 mM) treatment for 24 h with or without 666-15 pretreatment (1 μM) for 2 h (*n* = 6). GF, germfree rats receiving PBS gavage; GP, germfree rats receiving propionate gavage; GB, germfree rats receiving butyrate gavage; Pro, propionate; But, butyrate. * *p* < 0.05, ** *p* < 0.01, *** *p* < 0.001, according to one-way ANOVA with Tukey’s post-hoc test.

Transcriptional activation of the *Aanat* gene is an important mechanism for the induction of melatonin biosynthesis. To identify candidates that regulate *Aanat* transcription, we retrieved the potential transcription factors that could bind to the promoter region of the *Aanat* gene from the TRANSFAC (version 7.0) database [14]. A list of transcription factors was screened, as shown in Supplementary Table S1. In addition, related studies have suggested that melatonin synthesis involves protein kinase A-dependent phosphorylation of the transcription factor CREB and binding of p-CREB in the promoter region of the *Aanat* gene [15]. The results showed that the germfree rats receiving propionate gavage (GP) and germfree rats receiving butyrate gavage (GB) had higher p-CREB levels in the ileum and colon tissues than the GF group (*p* < 0.05), while the CREB level remained relatively unchanged (Figure 5A). When pretreated with the CREB inhibitor 666-15, AANAT level showed no significant elevation after treatment with propionate or butyrate (Figure 5B). Moreover, 666-15 also prevented the significant augmentation of melatonin resulting from propionate and butyrate treatment (Figure 5C).

### 3.5. Propionate and Butyrate Modulated the Metabolites and Related Metabolic Pathways

**Figure 6:**
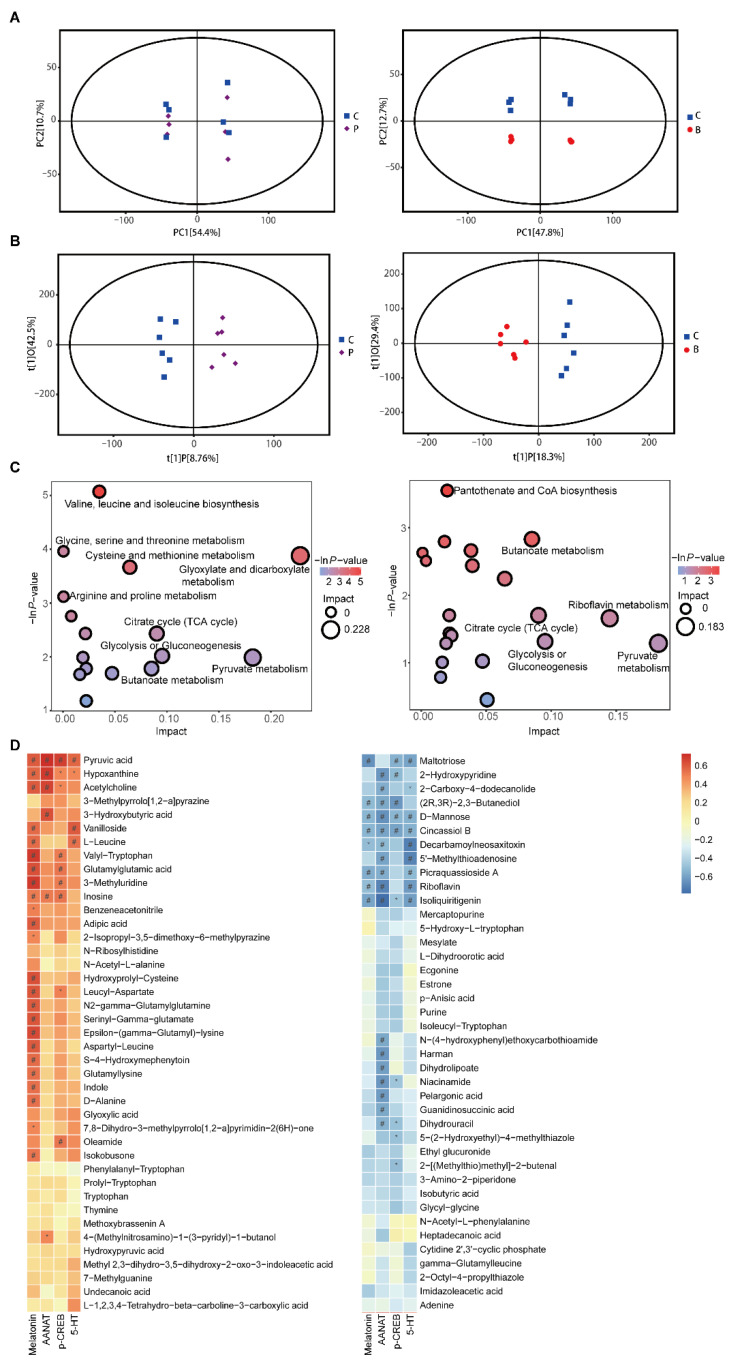
Metabolomics in BON-1 cell supernatant after propionate or butyrate treatment. (**A**) Score plots of the principal component analysis (PCA) model between the propionate group and the control group (left) and between the butyrate group and the control group (right). (**B**) Score plots of the orthogonal projections to latent structure-discriminant analysis (OPLS-DA) model between the propionate group and the control group (left) and between the butyrate group and the control group (right). (**C**) Bubble plots of pathway analysis between the propionate group and the control group (left) and between the butyrate group and the control group (right). (**D**) Correlation analysis between levels of different metabolites and those of melatonin, AANAT, p-CREB, and 5-HT in the supernatant of BON-1 cells. *, *p* < 0.05 and r ≥ 0.5; #, *p* < 0.05 and r < 0.5 (*n* = 6).

All the metabolites detected in positive and negative ion modes were merged and analyzed using SIMCA16.0.2 software. Principal component analysis (PCA) score plots indicated that butyrate treatment significantly influenced the metabolic profiling compared to the control group [R2X(cum) = 0.693], while the propionate group did not effectively separate from the control group [R2X(cum) = 0.73] (Figure 6A). Orthogonal projections to latent structure-discriminant analysis (OPLS-DA) with a better discriminative power than that of PCA was performed to characterize the metabolic profiles based on class information. OPLS-DA score plots showed clear clustering between the propionate group and control group [R2X(cum) = 0.512, R2Y(cum) = 0.951, Q2(cum) = 0.252], as well as between the butyrate group and control group [R2X(cum) = 0.477, R2Y(cum) = 0.944, Q2(cum) = 0.628] (Figure 6B).

The differentially expressed metabolites were screened according to variable importance in the projection (VIP) of the OPLS-DA model > 1 and *p* < 0.05. There were 43 compound IDs in the propionate group that were significantly different from those of the control group. Thirty-one compound IDs in the propionate group were upregulated, including hypoxanthine, pyruvic acid, D-alanine, and indole (Supplementary Table S2). There were 40 compound IDs in the butyrate group that were significantly different from those of the control group, and 10 compound IDs in the butyrate group were upregulated, such as pyruvic acid, hypoxanthine, and inosine (Supplementary Table S3). Then, pathway enrichment and topological analyses were performed based on differential metabolites in the BON-1 cell supernatant. Compared to control group, the following pathways were enriched in the propionate group, including amino acid metabolism pathways, such as “valine, leucine, and isoleucine biosynthesis”, “glycine, serine, and threonine metabolism”, and “tryptophan metabolism”; carbohydrate metabolism pathways, such as “citrate cycle”, “pyruvate metabolism”, and “butanoate metabolism”; nucleotide metabolism pathways, such as “purine metabolism”; and metabolism of other amino acids, such as “taurine and hypotaurine metabolism” (Figure 6C and Supplementary Table S4). The following pathways were enriched in the butyrate group: amino acid metabolism pathways, such as “glycine, serine, and threonine metabolism” and “valine, leucine, and isoleucine biosynthesis”; carbohydrate metabolism pathways, such as “butanoate metabolism” and “pyruvate metabolism”; and metabolism of other amino acids, such as “taurine and hypotaurine metabolism” (Figure 6C and Supplementary Table S5).

The relationships between metabolite levels and melatonin, AANAT, p-CREB, and 5-HT levels in the supernatant of BON-1 cells were analyzed. Correlation analysis demonstrated that the levels of various metabolites were significantly correlated with the levels of melatonin, AANAT, p-CREB, and 5-HT. Specifically, pyruvic acid was positively correlated with melatonin, AANAT, p-CREB, and 5-HT; inosine was positively correlated with melatonin, AANAT, and p-CREB; hypoxanthine and acetylcholine were positively correlated with melatonin and AANAT; and indole was positively correlated with melatonin, suggesting that these metabolites might promote melatonin synthesis (Figure 6D).

## 4. Discussion

In this study, we found that propionate and butyrate-producing bacteria, *R. hominis*, induced melatonin synthesis both in the intestinal mucosa and BON-1 cells. The underlying mechanisms involved promoting the 5-HT production and activating the p-CREB-AANAT pathway through propionate and butyrate.

Gut melatonin has multiple effects on the GI tract. Melatonin is a scavenger of free radicals and has antioxidant, immunomodulatory, and anti-inflammatory properties [16]. It can also regulate gastrointestinal motility and moderate visceral hypersensitivity [17]. When used in IBS treatment, melatonin shows the ability to improve abdominal pain and patient quality of life [18]. Moreover, melatonin adjuvant treatment reduced the severity of ulcerative colitis compared to a non-melatonin treatment group through exerting anti-inflammatory effects [19]. In a sleep-deprived mouse model with colonic mucosal injury and gut dysbiosis, melatonin supplementation improved mucosal injury and dysbiosis of the microbiota in the colon [20]. Our previous research revealed that melatonin cell density was positively correlated with the sensation thresholds of the urge to defecate and maximize tolerable distension in IBS-D patients, suggesting that increased melatonin levels may be a countermeasure for the development of IBS [4]. However, exogenous melatonin treatment for an extended period might cause adverse effects such as headache, rash, and nightmares [18]. Furthermore, previous studies have paid little attention to the effect of gut-derived melatonin on digestive diseases.

Melatonin and gut microbiota interact closely with each other. A study showed that melatonin levels significantly decreased in the sleep disorder group among children with autism spectrum disorder and was positively associated with the abundance of butyrate-producing bacteria, *Faecalibacterium* and *Agathobacter*. These previous findings imply that the reduction in gut melatonin levels might be elicited by a decrease in butyrate levels, which then potentially aggravates sleep problems and core autism symptoms in children with autism [21]. Therefore, finding a way to increase gut melatonin level might effectively improve sleep problems and some mental symptoms.

Gut melatonin levels can be influenced by food intake [7,22]. After consuming melatonin-rich foods, dietary melatonin may contribute to serum melatonin concentration [7]. In this study, germfree rats received the same kind of feed; thus, the influence of dietary factors on gut melatonin or microbiota could be excluded. In addition, no microorganisms were colonized in the germfree rats, which could eliminate the interference of other factors to the greatest extent possible. Therefore, germfree animals are ideal models for research on the relationship between the microbiota and the host. Our previous study illustrated that intestinal microbiota plays a pivotal role in colonic melatonin expression, and *Roseburia* abundance is positively related to the level of colonic mucosal melatonin [4]. In this study, we further confirmed that oral administration of *R. hominis* significantly increased intestinal melatonin levels, but not serum melatonin levels, in germfree rats (Figure 1 and Supplementary Figure S4). This finding suggests that treating germfree rats with a single strain, *R. hominis*, was insufficient to affect systemic melatonin levels.

*R. hominis* is a representative *Roseburia* species. Modification in *Roseburia* abundance may affect various metabolic pathways and is associated with several diseases, including IBS, ulcerative colitis, obesity, type 2 diabetes, nervous system disorders, and allergies [5,23]. Diets that are high in fermentable carbohydrates increase the relative abundance of *Roseburia*, which are capable of degrading polysaccharides, oligosaccharides, and sugars [24], indicating that fermentable carbohydrates might elevate the intestinal melatonin through increasing the abundance of *Roseburia*. *Roseburia* is characterized by SCFAs production, which can affect the intestinal epithelial barrier and regulate the function of innate immune cells through anti-inflammatory properties [25]. Our previous study suggested that gavage of *R. hominis* prevented stressed rats from developing visceral hypersensitivity and increased the propionate and butyrate levels in cecal contents [8]. In this study, SCFAs levels were measured in the intestinal contents after *R. hominis* treatment; thus, we found that propionate and butyrate levels significantly increased in both the cecum and colon (Figure 1). Some studies have demonstrated the promotional effects of SCFAs on intestinal hormone production such as 5-HT synthesized by ECs [6] and glucagon-like peptide-1 synthesized by L cells [26]. The above studies suggest that SCFAs might be key factors in the interactions between Roseburia and gut melatonin.

We further confirmed that both propionate and butyrate increased intestinal melatonin levels in germfree rats (Figure 2). Although propionate is less frequently studied compared to butyrate, it has some distinct health-promoting properties, such as lowering lipogenesis and serum cholesterol levels as well as anticarcinogenic effects [27]. Additionally, both propionate and butyrate can serve as inhibitors of histone deacetylases and can modulate gene expression through epigenetic regulation [28]. In this study, the distinction between the effects of propionate and butyrate on melatonin was not significant, indicating that propionate and butyrate might play a similar role in promoting melatonin synthesis.

An important upstream product in melatonin synthesis is 5-HT. A recent study showed that 5-HT levels decreased in germfree mice compared to those in SPF controls. The 5-HT level is elevated after exposure to bacterial metabolites, such as propionate and butyrate [6,29]. Melatonin and 5-HT are mainly synthesized by ECs. We confirmed that propionate and butyrate significantly increased the intestinal 5-HT and melatonin levels. Moreover, in cell experiments, pretreatment with the TPH inhibitor significantly reduced the promotional effect of propionate and butyrate on melatonin levels, suggesting that propionate and butyrate regulate melatonin synthesis by elevating 5-HT levels (Figure 3). Metabolomics analysis showed that propionate increased the indole level in BON-1 cell supernatant, and indole level was positively related to melatonin levels (Figure 6). Both 5-HT and melatonin are derivatives of indole; they are involved in the tryptophan metabolism pathway, which was enriched in the propionate group. Therefore, propionate might increase melatonin synthesis by activating tryptophan metabolism, which requires further verification.

**Figure 7:**
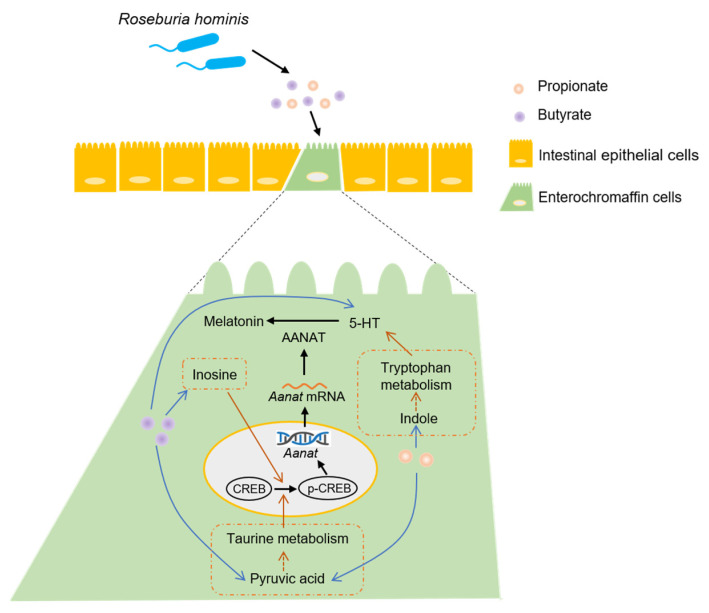
Potential mechanisms of *Roseburia hominis* in intestinal melatonin synthesis.

Some researchers have reported the relationship between butyrate and melatonin synthesis. Baumann et al. [30] found that supplementation with sodium butyrate improved gut melatonin synthesis by elevating hydroxyindole-O-methyltransferase (an enzyme of melatonin synthesis) levels in a nonalcoholic steatohepatitis mouse model. Another study found that treatment with sodium butyrate increased the melatonin level in duodenal tissue and Caco-2 cells in a dose- and time-dependent manner [31]. AANAT is a vital rate-limiting enzyme in melatonin biosynthesis. Therefore, we screened a list of transcription factors for *Aanat*. In addition, related studies have suggested that CREB is an important transcription factor and that its phosphorylation could regulate *Aanat* expression, leading to the activation of melatonin synthesis [17]. A study showed that colonization of gut microbiota in germfree mice caused the level of CREB to increase and that of p-CREB to decrease in the hippocampus, suggesting an important role of microbiota in modulating CREB expression and relevant signaling [32]. The novel probiotic *Bifidobacterium longum* subsp. *infantis* strain CCFM687 significantly upregulated CREB gene expression in the prefrontal cortex of the brain in a chronic stress-induced depression mouse model [33]. According to our results, propionate and butyrate treatment significantly increased the levels of AANAT and p-CREB both in vivo and in vitro (Figure 4 and Figure 5). Furthermore, pretreatment of BON-1 cells with *Aanat* siRNA or p-CREB inhibitor before treatment with propionate or butyrate led to a significant reduction in the concentration of melatonin in the cell supernatant. This finding demonstrates that propionate and butyrate regulate melatonin synthesis by activating the p-CREB-AANAT pathway. Metabolomics analysis demonstrated that pyruvic acid levels were significantly elevated after treatment with propionate or butyrate in BON-1 cells. Furthermore, pyruvic acid levels were positively related to melatonin, AANAT, p-CREB, and 5-HT levels. Pyruvic acid participates in taurine metabolism, which was enriched in both propionate and butyrate groups (Figure 6). A previous study found that taurine supplementation significantly increased the p-CREB level in the hippocampus and improved the depressive behaviors of rats [34], suggesting that propionate and butyrate might activate p-CREB-AANAT signaling and promote melatonin synthesis by enhancing taurine metabolism. In addition, butyrate treatment significantly increased the inosine level in the BON-1 cell supernatant. Animal experiments have reported that inosine injection significantly increases p-CREB levels in the hippocampus and prefrontal cortex of rats [35]. The correlation analysis demonstrated a positive relationship between inosine level and melatonin, AANAT, and p-CREB levels, suggesting that inosine might promote melatonin synthesis by increasing p-CREB and AANAT levels, which requires further investigation (Figure 7). Other studies have reported potential mechanisms between butyrate and CREB. Butyrate was found to upregulate phosphatidylinositol 3-kinase (PI3K) expression, which in turn enhanced the phosphorylation of its downstream effector, CREB, in BV-2 cells (a murine microglial cell line) owing to the role of histone modification of butyrate [36]. In another study, butyrate increased cAMP levels and PKA activity and induced CREB phosphorylation in Caco-2 cells through increased ATP production, suggesting that butyrate might promote AANAT and melatonin synthesis by activating cAMP-PKA-CREB signaling, which requires further research [37].

## 5. Couclusions

In conclusion, *R. hominis* increased intestinal melatonin synthesis through its metabolites, propionate and butyrate. The underlying mechanism was that propionate and butyrate promoted 5-HT synthesis and activated the p-CREB-AANAT pathway. Our study provides novel evidence that microbiota intervention could be a potential therapeutic strategy for some intestinal diseases, such as IBS and inflammatory bowel disease, by increasing intestinal melatonin levels.

## Data Availability

The data presented in this study are available on request from the corresponding author.

## Supplementary Materials

The following supporting information can be downloaded at: https://www.mdpi.com/article/10.3390/nu14010117/s1, Figure S1: Concentration of acetate, isobutyrate and isovalerate in intestinal contents detected using gas chromatography-mass spectrometry after gavage of *Roseburia hominis*. Figure S2: Propionate and butyrate alleviated the visceral hypersensitivity in germfree rats. Figure S3: Effects of propionate and butyrate on AANAT level in BON-1 cells. Figure S4: Serum melatonin level in response to oral administration of *Roseburia hominis*, propionate, or butyrate. Table S1: Possible transcription factors of *Aanat*. Table S2: Differentially expressed metabolites between propionate group and control group. Table S3: Differentially expressed metabolites between butyrate group and control group. Table S4: Pathway analysis between propionate group and control group. Table S5: Pathway analysis between butyrate group and control group.

## References

[1] Acuna-Castroviejo D., Escames G., Venegas C., Diaz-Casado M.E., Lima-Cabello E., Lopez L.C., Rosales-Corral S., Tan D.X., Reiter R.J. (2014). Extrapineal melatonin: Sources, regulation, and potential functions. Cell. Mol. Life Sci..

[2] Esteban-Zubero E., Lopez-Pingarron L., Alatorre-Jimenez M.A., Ochoa-Moneo P., Buisac-Ramon C., Rivas-Jimenez M., Castan-Ruiz S., Antonanzas-Lombarte A., Tan D.X., Garcia J.J. (2017). Melatonin’s role as a co-adjuvant treatment in colonic diseases: A review. Life Sci..

[3] Andersen L.P., Gogenur I., Rosenberg J., Reiter R.J. (2016). The safety of melatonin in humans. Clin. Drug Investig..

[4] Wang B., Zhu S., Liu Z., Wei H., Zhang L., He M., Pei F., Zhang J., Sun Q., Duan L. (2020). Increased expression of colonic mucosal melatonin in patients with irritable bowel syndrome correlated with gut dysbiosis. Genom. Proteom. Bioinform..

[5] Tamanai-Shacoori Z., Smida I., Bousarghin L., Loreal O., Meuric V., Fong S.B., Bonnaure-Mallet M., Jolivet-Gougeon A. (2017). *Roseburia* spp.: A marker of health?. Future Microbiol..

[6] Yano J.M., Yu K., Donaldson G.P., Shastri G.G., Ann P., Ma L., Nagler C.R., Ismagilov R.F., Mazmanian S.K., Hsiao E.Y. (2015). Indigenous bacteria from the gut microbiota regulate host serotonin biosynthesis. Cell.

[7] Ma N., Zhang J., Reiter R.J., Ma X. (2020). Melatonin mediates mucosal immune cells, microbial metabolism, and rhythm crosstalk: A therapeutic target to reduce intestinal inflammation. Med. Res. Rev..

[8] Zhang J., Song L., Wang Y., Liu C., Zhang L., Zhu S., Liu S., Duan L. (2019). Beneficial effect of butyrate-producing Lachnospiraceae on stress-induced visceral hypersensitivity in rats. J. Gastroenterol. Hepatol..

[9] Liao X., Song L., Zeng B., Liu B., Qiu Y., Qu H., Zheng Y., Long M., Zhou H., Wang Y. (2019). Alteration of gut microbiota induced by DPP-4i treatment improves glucose homeostasis. EBioMedicine.

[10] Herrera-Martínez A.D., Feelders R.A., Van den Dungen R., Dogan-Oruc F., van Koetsveld P.M., Castaño J.P., de Herder W.W., Hofland L.J. (2020). Effect of the tryptophan hydroxylase inhibitor telotristat on growth and serotonin secretion in 2D and 3D cultured pancreatic neuroendocrine tumor cells. Neuroendocrinology.

[11] Zhang B., Zhang P., Tan Y., Feng P., Zhang Z., Liang H., Duan W., Jin Z., Wang X., Liu J. (2019). C1q-TNF-related protein-3 attenuates pressure overload-induced cardiac hypertrophy by suppressing the p38/CREB pathway and p38-induced ER stress. Cell Death Dis..

[12] Yu L., Lai Q., Feng Q., Li Y., Feng J., Xu B. (2021). Serum metabolic profiling analysis of chronic gastritis and gastric cancer by untargeted metabolomics. Front. Oncol..

[13] Matthes S., Bader M. (2018). Peripheral serotonin synthesis as a new drug target. Trends Pharmacol. Sci..

[14] Li M., Wu J., Hu G., Song Y., Shen J., Xin J., Li Z., Liu W., Dong E., Xu M. (2021). Pathological matrix stiffness promotes cardiac fibroblast differentiation through the POU2F1 signaling pathway. Sci. China Life Sci..

[15] Schomerus C., Korf H.W. (2005). Mechanisms regulating melatonin synthesis in the mammalian pineal organ. Ann. N. Y. Acad. Sci..

[16] Yin J., Li Y., Han H., Ma J., Liu G., Wu X., Huang X., Fang R., Baba K., Bin P. (2020). Administration of exogenous melatonin improves the diurnal rhythms of the gut microbiota in mice fed a high-fat diet. mSystems.

[17] Cipolla-Neto J., Amaral F.G.D. (2018). Melatonin as a hormone: New physiological and clinical insights. Endocr. Rev..

[18] Siah K.T., Wong R.K., Ho K.Y. (2014). Melatonin for the treatment of irritable bowel syndrome. World J. Gastroenterol..

[19] Chojnacki C., Wisniewska-Jarosinska M., Walecka-Kapica E., Klupinska G., Jaworek J., Chojnacki J. (2011). Evaluation of melatonin effectiveness in the adjuvant treatment of ulcerative colitis. J. Physiol. Pharmacol..

[20] Gao T., Wang Z., Dong Y., Cao J., Lin R., Wang X., Yu Z., Chen Y. (2019). Role of melatonin in sleep deprivation-induced intestinal barrier dysfunction in mice. J. Pineal Res..

[21] Hua X., Zhu J., Yang T., Guo M., Li Q., Chen J., Li T. (2020). The gut microbiota and associated metabolites are altered in sleep disorder of children with Autism spectrum disorders. Front. Psychiatry.

[22] Yasmin F., Sutradhar S., Das P., Mukherjee S. (2021). Gut melatonin: A potent candidate in the diversified journey of melatonin research. Gen. Comp. Endocrinol..

[23] Machiels K., Joossens M., Sabino J., De Preter V., Arijs I., Eeckhaut V., Ballet V., Claes K., Van Immerseel F., Verbeke K. (2014). A decrease of the butyrate-producing species *Roseburia hominis* and *Faecalibacterium prausnitzii* defines dysbiosis in patients with ulcerative colitis. Gut.

[24] Tomova A., Bukovsky I., Rembert E., Yonas W., Alwarith J., Barnard N.D., Kahleova H. (2019). The effects of vegetarian and vegan diets on gut microbiota. Front. Nutr..

[25] Yao Y., Cai X., Fei W., Ye Y., Zhao M., Zheng C. (2020). The role of short-chain fatty acids in immunity, inflammation and metabolism. Crit. Rev. Food Sci. Nutr..

[26] Yadav H., Lee J.H., Lloyd J., Walter P., Rane S.G. (2013). Beneficial metabolic effects of a probiotic via butyrate-induced GLP-1 hormone secretion. J. Biol. Chem..

[27] Hosseini E., Grootaert C., Verstraete W., Van de Wiele T. (2011). Propionate as a health-promoting microbial metabolite in the human gut. Nutr. Rev..

[28] Kasubuchi M., Hasegawa S., Hiramatsu T., Ichimura A., Kimura I. (2015). Dietary gut microbial metabolites, short-chain fatty acids, and host metabolic regulation. Nutrients.

[29] Reigstad C.S., Salmonson C.E., Rainey J.F., Szurszewski J.H., Linden D.R., Sonnenburg J.L., Farrugia G., Kashyap P.C. (2015). Gut microbes promote colonic serotonin production through an effect of short-chain fatty acids on enterochromaffin cells. FASEB J..

[30] Baumann A., Jin C.J., Brandt A., Sellmann C., Nier A., Burkard M., Venturelli S., Bergheim I. (2020). Oral supplementation of sodium butyrate attenuates the progression of non-alcoholic steatohepatitis. Nutrients.

[31] Jin C.J., Engstler A.J., Sellmann C., Ziegenhardt D., Landmann M., Kanuri G., Lounis H., Schröder M., Vetter W., Bergheim I. (2016). Sodium butyrate protects mice from the development of the early signs of non-alcoholic fatty liver disease: Role of melatonin and lipid peroxidation. Br. J. Nutr..

[32] Zeng L., Zeng B., Wang H., Li B., Huo R., Zheng P., Zhang X., Du X., Liu M., Fang Z. (2016). Microbiota modulates behavior and protein kinase C mediated cAMP response element-binding protein signaling. Sci. Rep..

[33] Tian P., Zou R., Song L., Zhang X., Jiang B., Wang G., Lee Y.K., Zhao J., Zhang H., Chen W. (2019). Ingestion of *Bifidobacterium longum* subspecies infantis strain CCFM687 regulated emotional behavior and the central BDNF pathway in chronic stress-induced depressive mice through reshaping the gut microbiota. Food Funct..

[34] Toyoda A., Iio W. (2013). Antidepressant-like effect of chronic taurine administration and its hippocampal signal transduction in rats. Adv. Exp. Med. Biol..

[35] Yuan S., Jiang X., Zhou X., Zhang Y., Teng T., Xie P. (2018). Inosine alleviates depression-like behavior and increases the activity of the ERK-CREB signaling in adolescent male rats. Neuroreport.

[36] Saw G., Krishna K., Gupta N., Soong T.W., Mallilankaraman K., Sajikumar S., Dheen S.T. (2020). Epigenetic regulation of microglial phosphatidylinositol 3-kinase pathway involved in long-term potentiation and synaptic plasticity in rats. Glia.

[37] Wang A., Si H., Liu D., Jiang H. (2012). Butyrate activates the cAMP-protein kinase A-cAMP response element-binding protein signaling pathway in Caco-2 cells. J. Nutr..

