# Supplementary material for: Roseburia hominis Increases Intestinal Melatonin Level by Activating p-CREB-AANAT Pathway
Source: Nutrients. 2021 Dec 28;14(1):117. doi: 10.3390/nu14010117 (PMC8746519; doi:10.3390/nu14010117)
Supplement: Supplementary file 1 [file nutrients-14-00117-s001.zip › Supplementary Figure 1-4.pdf]

Supplementary Figures

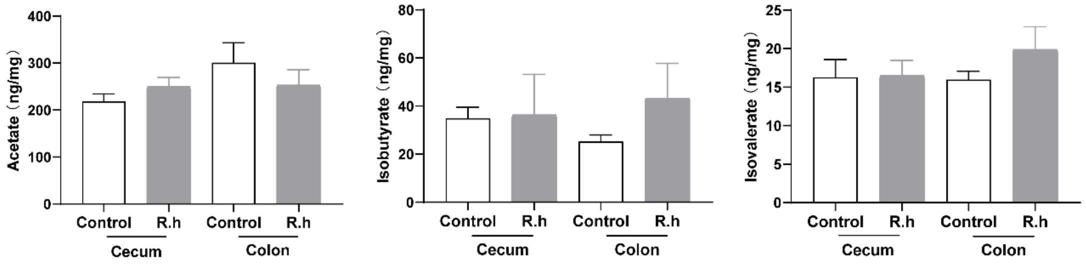

Supplementary Figure S1. Concentration of acetate, isobutyrate and isovalerate in intestinal contents detected using gas chromatography-mass spectrometry after gavage of *Roseburia hominis*. Concentrations of acetate, isobutyrate and isovalerate in cecal and colonic contents of germfree rats after gavage of PBS or *R. hominis* (R.h, 2×10^9 CFU/day) for 5 days. Analysis was performed using unpaired Student's *t*-test (n = 6).

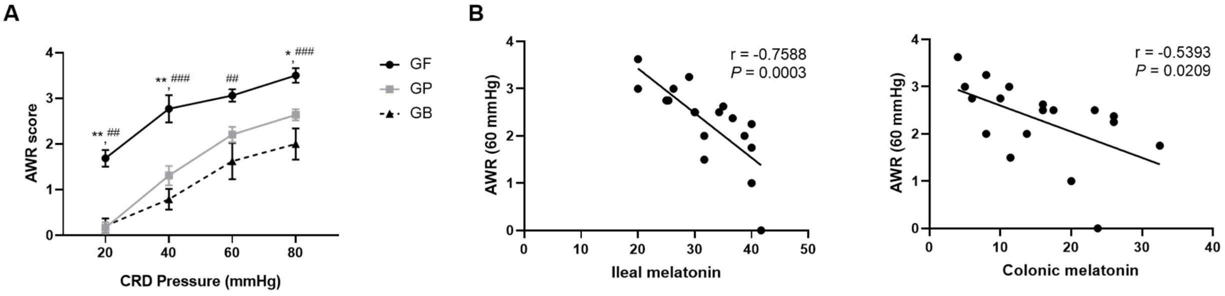

Supplementary Figure S2. Propionate and butyrate alleviated the visceral hypersensitivity in germfree rats. (**A**) The comparison of AWR scores among GF, GP and GB rats. Analysis was performed using One-way ANOVA with Dunnett's post-hoc test (n = 6). GF, germ-free rats received PBS gavage; GP, germfree rats received propionate gavage; GB, germfree rats received butyrate gavage. *, GF *vs.* GP, $^{*}P < 0.05$, $^{**}P < 0.01$; $^{\#}$, GF *vs.* GB, $^{\#\#}P < 0.01$, $^{\#\#\#}P < 0.001$. (**B**) The correlation between intestinal (ileum and colon) melatonin and AWR score at 60 mmHg. Analysis was performed using Pearson correlation test. AWR, abdominal withdrawal reflex. CRD, colorectal distension.

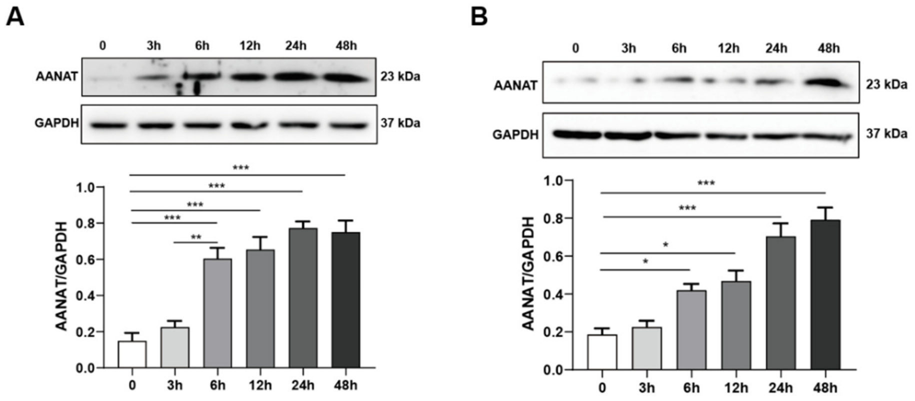

Supplementary Figure S3. Effects of propionate and butyrate on AANAT level in BON-1 cells. (**A**) Representative images (above) and quantification of AANAT level (below) in BON-1 cells after propionate (10 mM) treatment in different points of time. (**B**) Representative images (above) and quantification of AANAT level (below) in BON-1 cells after butyrate (10 mM) treatment in different points of time. Results were obtained using western blotting and analyzed using one-way ANOVA with Tukey's post-hoc test (n = 4). **P* < 0.05, ***P* <0.01, ****P* < 0.001.

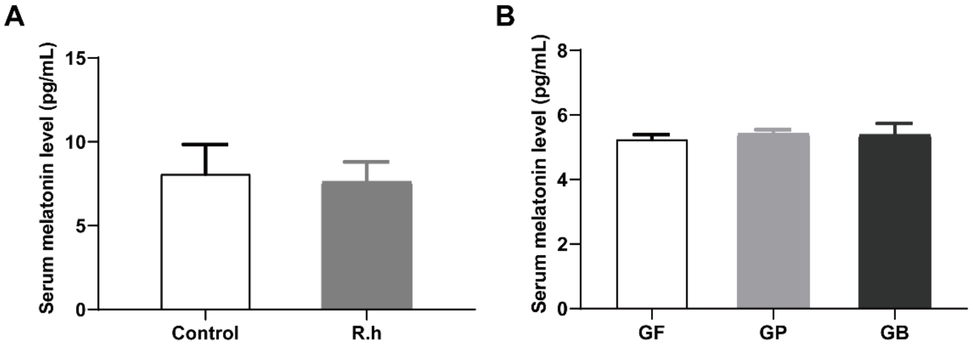

Supplementary Figure S4. Serum melatonin level in response to oral administration of *Roseburia hominis*, propionate, or butyrate. (A) Serum melatonin level in germfree rats after gavage with phosphate-buffered saline (PBS) or *R. hominis* (R.h, 2 × 10^9 CFU/day) for 5 days. Analysis was performed using unpaired Student's t-test (n = 6). (B) Serum melatonin level in germfree rats after gavage with PBS, sodium propionate, or sodium butyrate (300 mg/kg/day) for 7 days. Analysis was performed using one-way ANOVA with Tukey's post-hoc test (n = 6). GF, germfree rats received PBS gavage; GP, germfree rats received propionate gavage; GB, germfree rats received butyrate gavage.
